# Supplementary material for: Multi-strain analysis of Pseudomonas putida reveals the metabolic and genetic diversity of the species
Source: mSystems. 2026 Apr 1;11(4):e01594-25. doi: 10.1128/msystems.01594-25 (PMC13098254; doi:10.1128/msystems.01594-25)
Supplement: Supplemental figures — Figures S1 to S9. [file msystems.01594-25-s0001.docx]

**Supplemental Information**

Multi-strain Analysis of *Pseudomonas putida* Reveals Metabolic and Genetic Diversity of the Species

Joshua Mueller^1,4#^, Jayanth Krishnan^2#^, Qixing Wei^1^, Ying Hefner^2^, Jonathan M. Monk^2^, Hans Verkler^2^, Juan D. Tibocha-Bonilla^6+^, Anthony Ayala^3^, Bernhard O. Palsson^2,4,5^, Adam M. Feist^2,4,5^*, and Wei Niu^1^*

^1^ Department of Chemical and Biomolecular Engineering, University of Nebraska-Lincoln, Lincoln, NE, 68588, United States

^2^ Department of Bioengineering, University of California, San Diego, La Jolla, CA, 92093, United States

^3^ Department of Chemical Engineering, University of Puerto Rico-Mayagüez, Mayagüez, 00680, Puerto Rico

^4^ Joint BioEnergy Institute, 5885 Hollis Street, 4th Floor, Emeryville, CA, 94608, United States

^5^ Novo Nordisk Foundation Center for Biosustainability, Technical University of Denmark, 2800, Kgs, Lyngby, Denmark

^6^ Department of Pediatrics, University of California, San Diego, CA 92093-0760

^+^ Current address: Biological Systems and Engineering Division, Lawrence Berkeley National

Laboratory, Berkeley, CA, 94720, USA

^#^These authors contributed equally to this work.

*To whom correspondence should be addressed. [afeist@ucsd.edu](mailto:afeist@ucsd.edu), [wniu2@unl.edu](mailto:wniu2@unl.edu)

**Table of Contents**

Table S1. Whole-genome sequencing and assembly of 40 ATCC strains 3

Table S2. Pairwise distance matrix 3

Table S3**.** Metabolic data from the Biolog experiment 3

Table S4. List of all strains in pangenome analysis 3

Table S5. Growth prediction by 24 strain-specific GEMs 3

Table S6. Summary of MEMOTE reports of strain-specific models 3

Table S7. Clustering of strains based on CD-HIT analysis 3

Table S8. Bi-directional BLAST Hit (BBH) table 3

Table S9. High-frequency alleles in alleleome analysis 3

Table S10. Phylogenetic analysis of alleles 3

Table S11. Substrate grouping for phenotypic analysis 3

Figure S1. Wet-lab experiments for reconstructing Pan-putida metabolic network 4

Figure S2. Agar plate growth assay on aromatic compounds 5-6

Figure S3. Phenotypic data analysis of 42 *E. coli* strains 7

Figure S4. Metabolic model reconstruction 8

Figure S5. Strain-specific GEM construction 9-10

Figure S6. Alignment analysis of CsrA and Crc alleles 11

Figure S7. Major and peripheral pathways of aromatics metabolism in alleleome analysis 12

Figure S8. Alleleome analysis of pathway enzymes in aromatics metabolism 13

Figure S9. Phylogenetic analysis of pathway enzymes in aromatics metabolism 14-19

# **Following Supplemental Materials are submitted as separate Excel files.**

# Table S1. Whole-genome sequencing and assembly of 40 ATCC strains

Table S2. Pairwise distance matrix

Table S3**.** Metabolic data from the Biolog experiment

Table S4. List of all strains in pangenome analysis

Table S5. Growth prediction by 24 strain-specific GEMs

Table S6. Summary of MEMOTE reports of strain-specific models

Table S8. High-frequency alleles in alleleome analysis

Table S9. Phylogenetic analysis of alleles

Table S10. Substrate grouping for phenotypic analysis

Table S11. BiGG IDs for additional aromatic substrates.

# **Following Supplemental Materials are available at** [**https://doi.org/10.5281/zenodo.18715116**](https://doi.org/10.5281/zenodo.18715116) **due to their large file size.**

Table S7. Clustering of strains based on CD-HIT analysis

Table S12. Bi-directional BLAST Hit (BBH) table

**Genome data.** Assembly fasta files of 40 strains have been submitted to NCBI under the BioProject ID PRJNA1347105 and are additionally available at <https://doi.org/10.5281/zenodo.18488648>. Genome annotation and quality report are deposited in <https://doi.org/10.5281/zenodo.17382094>.

**Phenotypic microarray data.** All Biolog phenotype microarray data is available for visualization and parsing on PMKbase.com and <https://doi.org/10.5281/zenodo.17382094>.

**GEM Model data.** The updated metabolic model, strain specific models and MEMOTE reports can be found on <https://doi.org/10.5281/zenodo.17382094,>

**
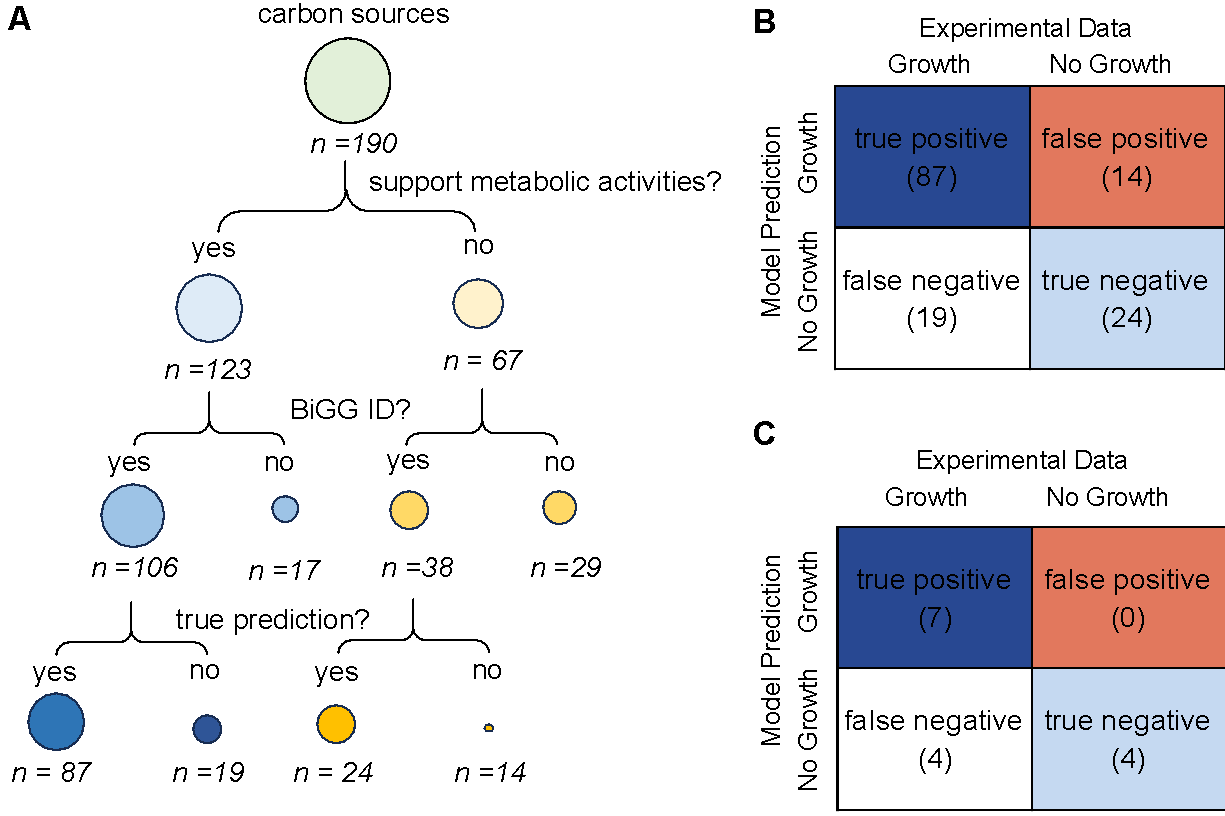
**

**Figure S1.** Wet-lab experiments for reconstructing Pan-putida metabolic network. **A.** Summary of how Biolog data was incorporated into the model construction. **B.** Agreement of Pan-putida draft model prediction and Biolog data. **C.** Agreement of Pan-putida draft model prediction and aromatics growth data.

**
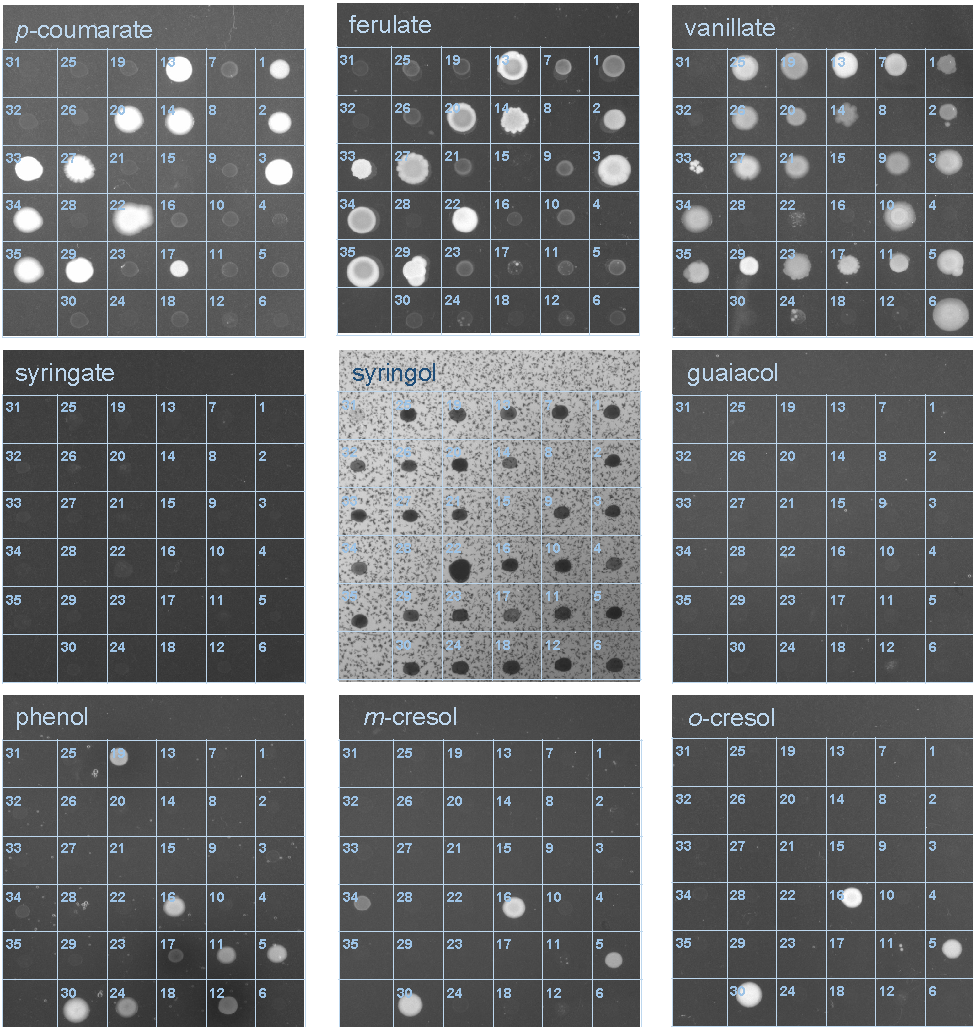
**

**
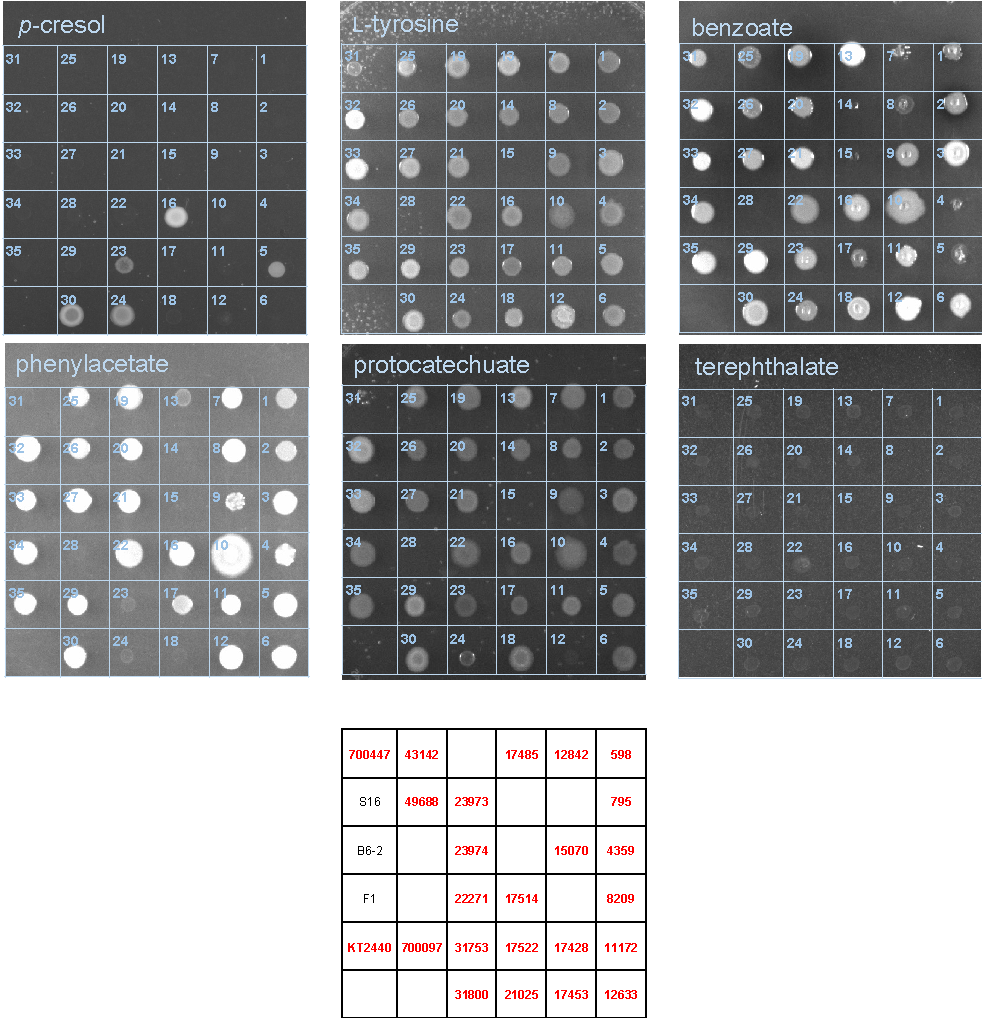
**

**Figure S2.** Images of agar plate growth assays on aromatic compounds. The legend for each plate is on the top of the figure, strains that were also characterized in the Biolog assays are in Red.


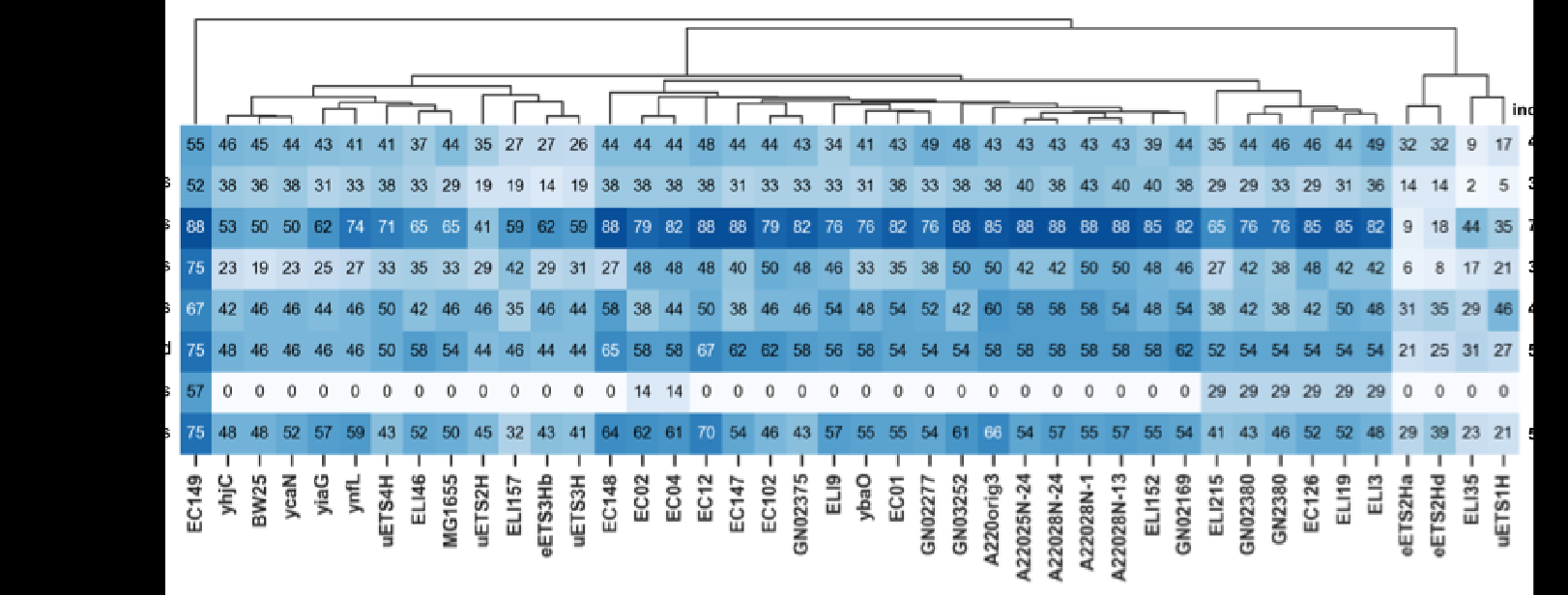


**Figure S3.** Phenotypic data analysis of 42 *E. coli* strains. Clustered heatmap showing metabolic activity profiles of 42 *E. coli* across 205 carbon substrates, including 190 Biolog compounds and 15 additional aromatic compounds. Each row corresponds to a compound group (group numbers defined in the main text) and each column represents a strain. The numerical value within each cell indicates the percentage of compounds in the corresponding group on which the strain exhibited metabolic activity. Color intensity is directly scaled to the numerical values, with darker shades representing higher percentage. Font color was adjusted to enhance readability. Metabolic activity index values for *E. coli* species on each compound group are shown on the right.

**
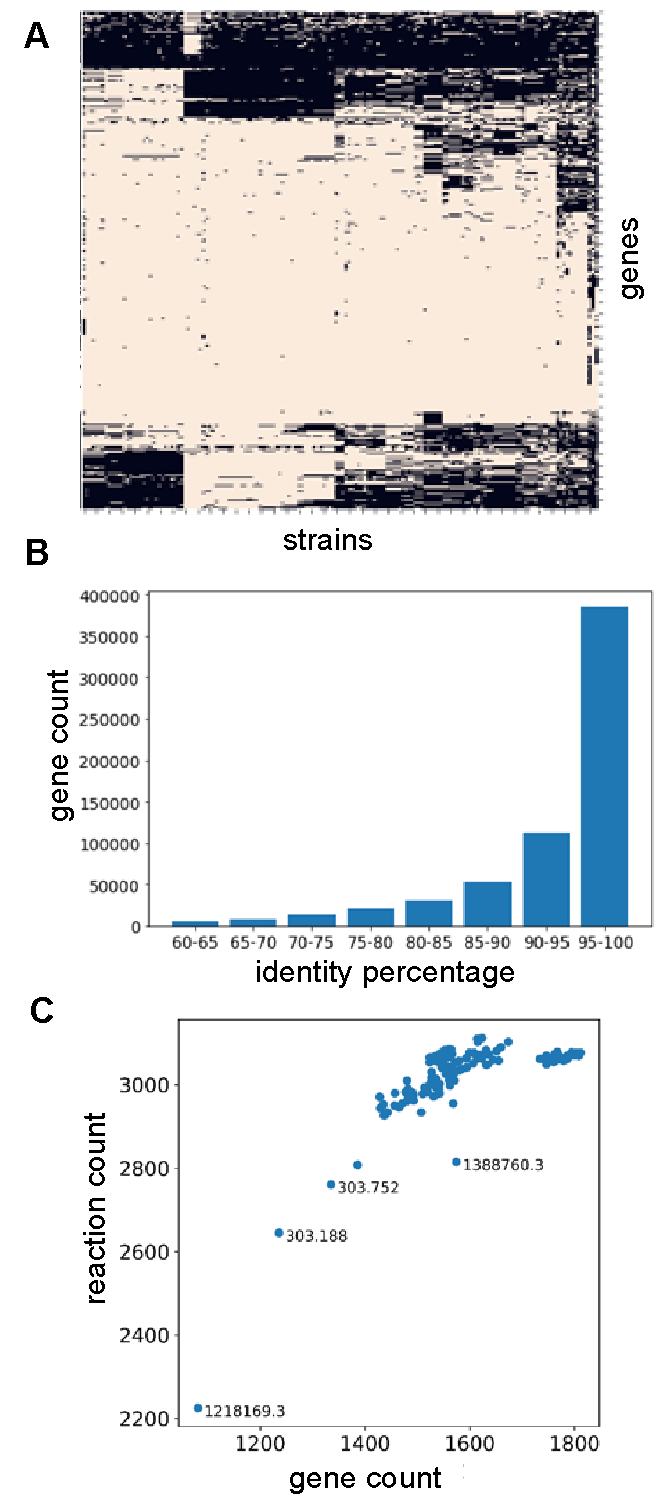
**


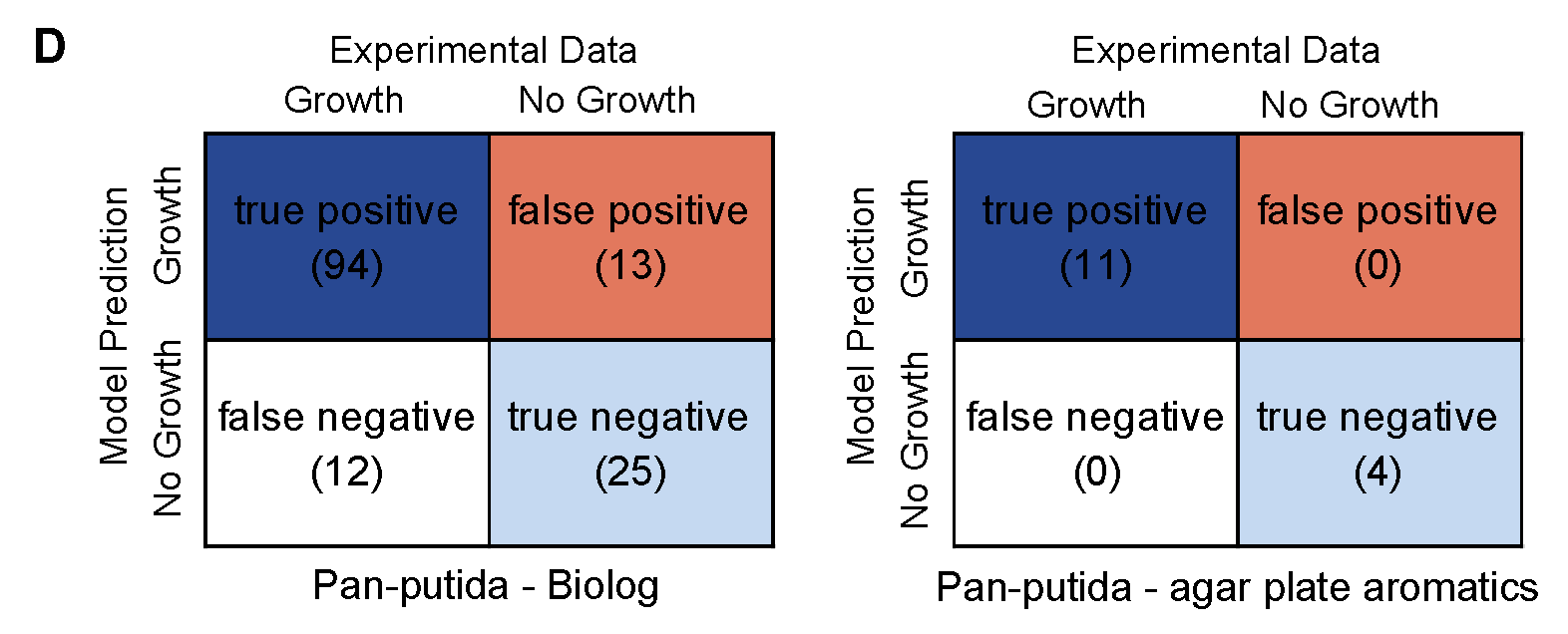


**Figure S4.** Metabolic model reconstruction. **A.** Core-genome analysis. Comparison of gene presence(white)/absence(black) for each of the 164 strains. **B.** Pan-putida BBH hits sequence identities. The number of homologous genes identified during the BBH analysis with a sequence identity in the given range. **C.** Model gene and reaction count for strain-specific model. A scatterplot of the number of genes and reactions contained in each strain-specific model. The models are largely clustered in the top right corner which indicates significant shared metabolic content across the species. **D.** Final model prediction accuracy. Agreement of Pan-putida model prediction with Biolog data and agar plate-based growth experiments. Color codes: dark blue, true positive; light blue, true negative; orange, false positive; white, false negative.

**
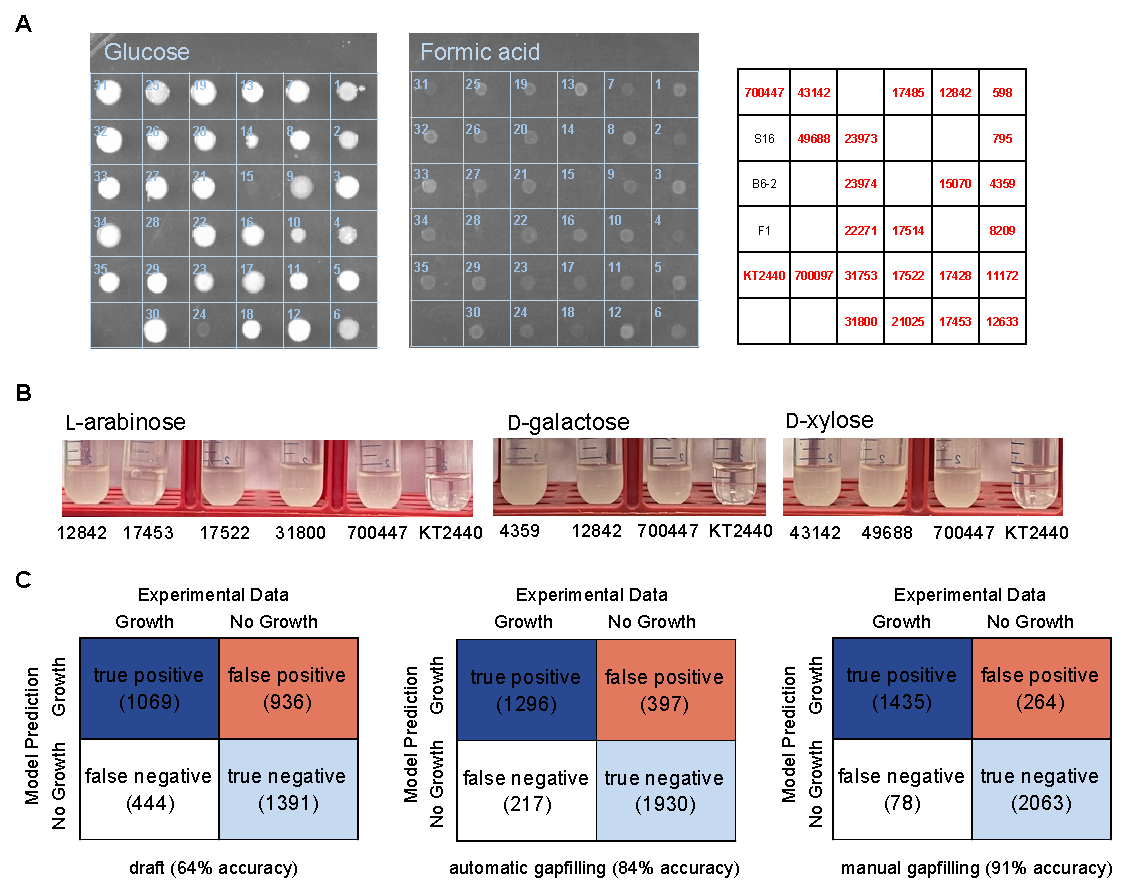
**

**
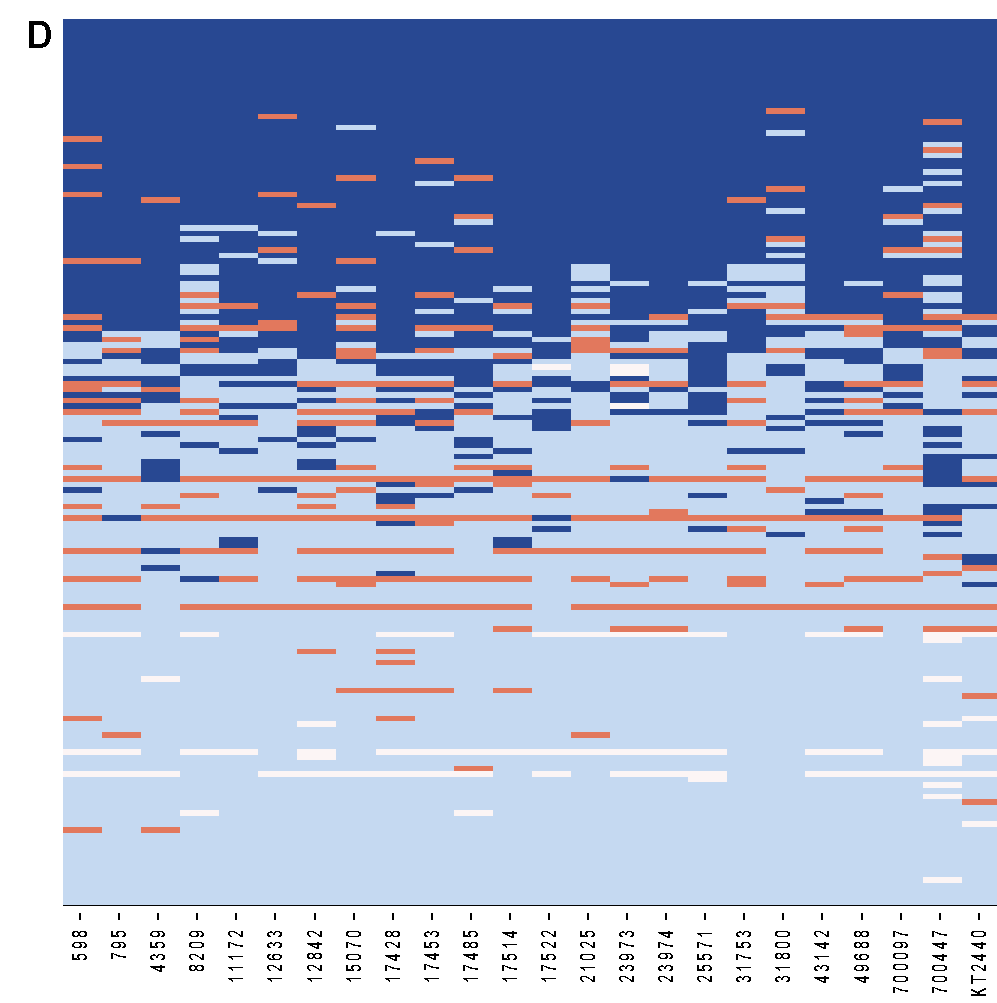
**

**Figure S5.** Strain-specific GEM construction. **A.** Culturing on agar plates containing indicated compound as the sole carbon source. A grid legend is provided. GEMs were constructed for strains in bold. **B.** Culturing of selected strains in liquid media containing the indicated monosaccharide as the sole carbon source. **C.** Refinement of strain-specific GEMs. **D.** Comparison of model-specific predictions to experimental results. Each column represents a specific strain, and each row represents a carbon source. Color codes: dark blue, true positive; light blue, true negative; orange, false positive; white, false negative.

**
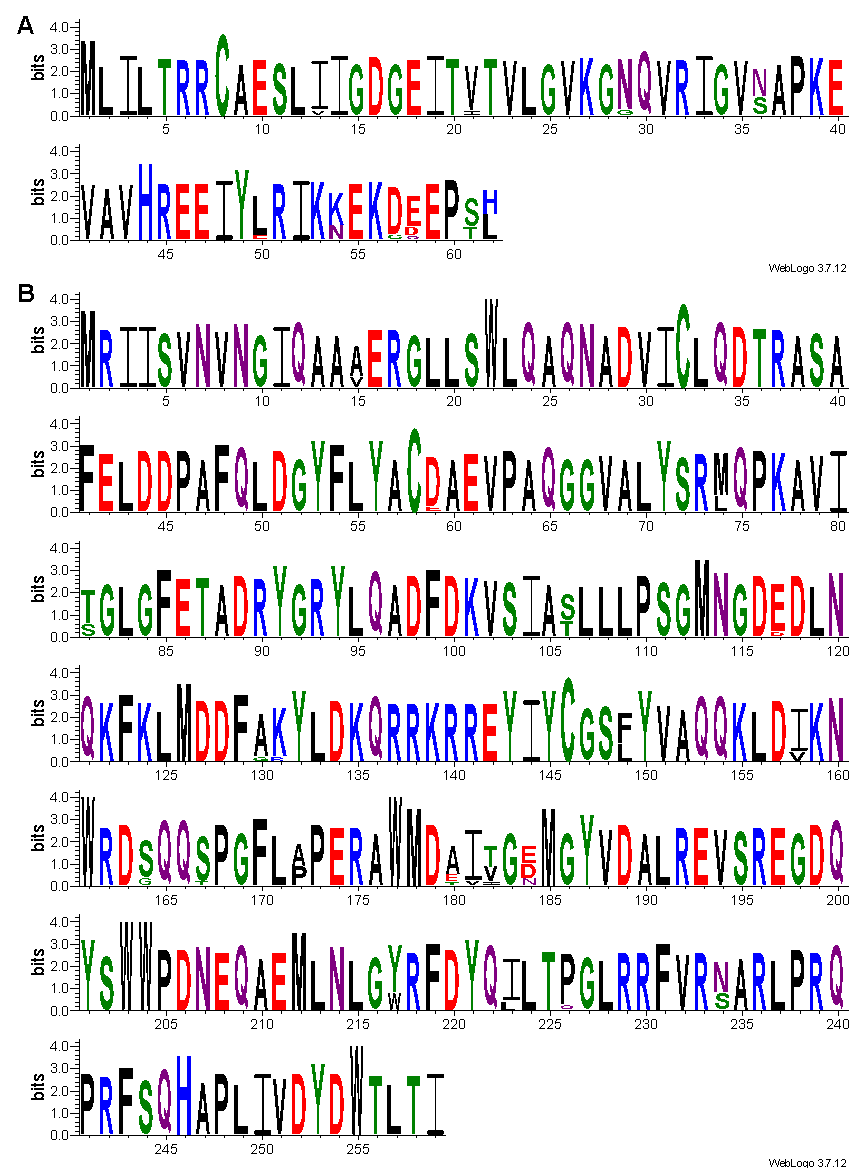
**

**Figure S6.** Consensus sequence analysis of CsrA (**A**) and Crc (**B**) alleles. The results are presented using the WebLogo program (https://weblogo.berkeley.edu/logo.cgi).


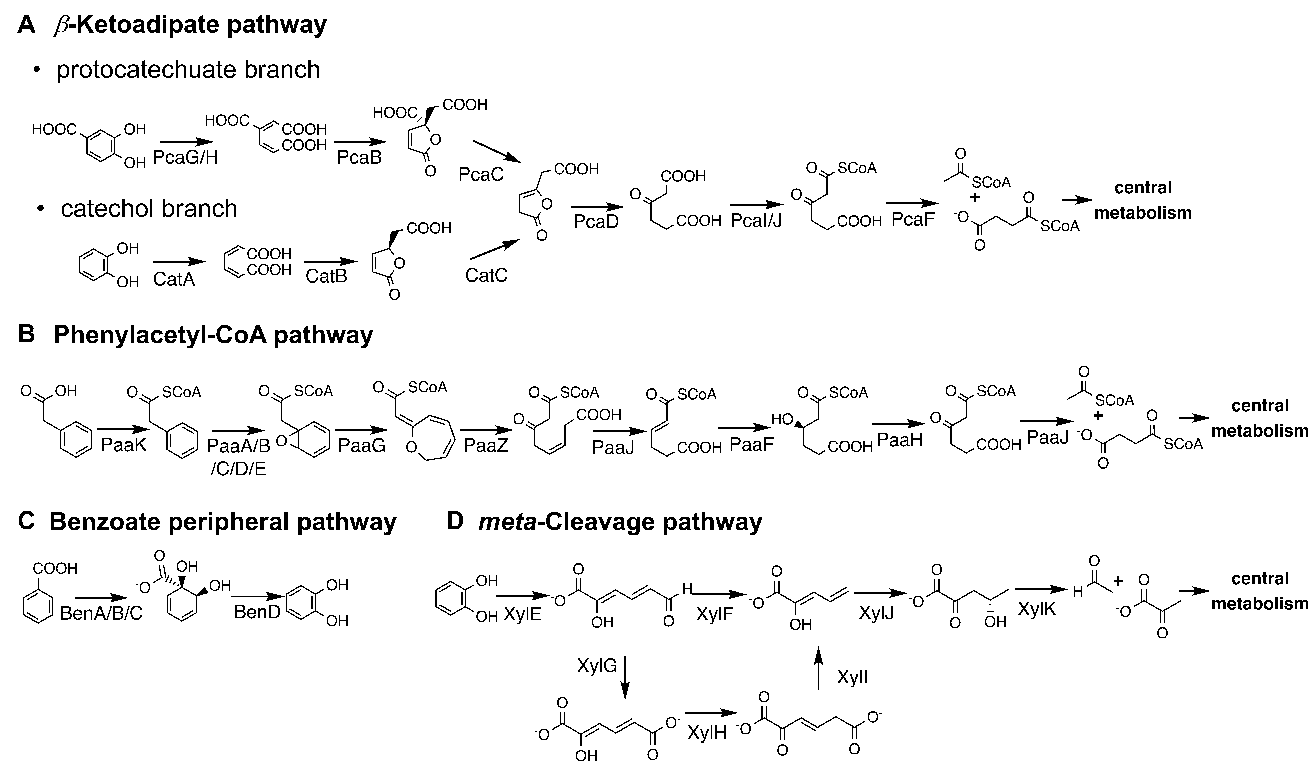


**Figure S7.** Major and peripheral pathways of aromatics metabolism in alleleome analysis.


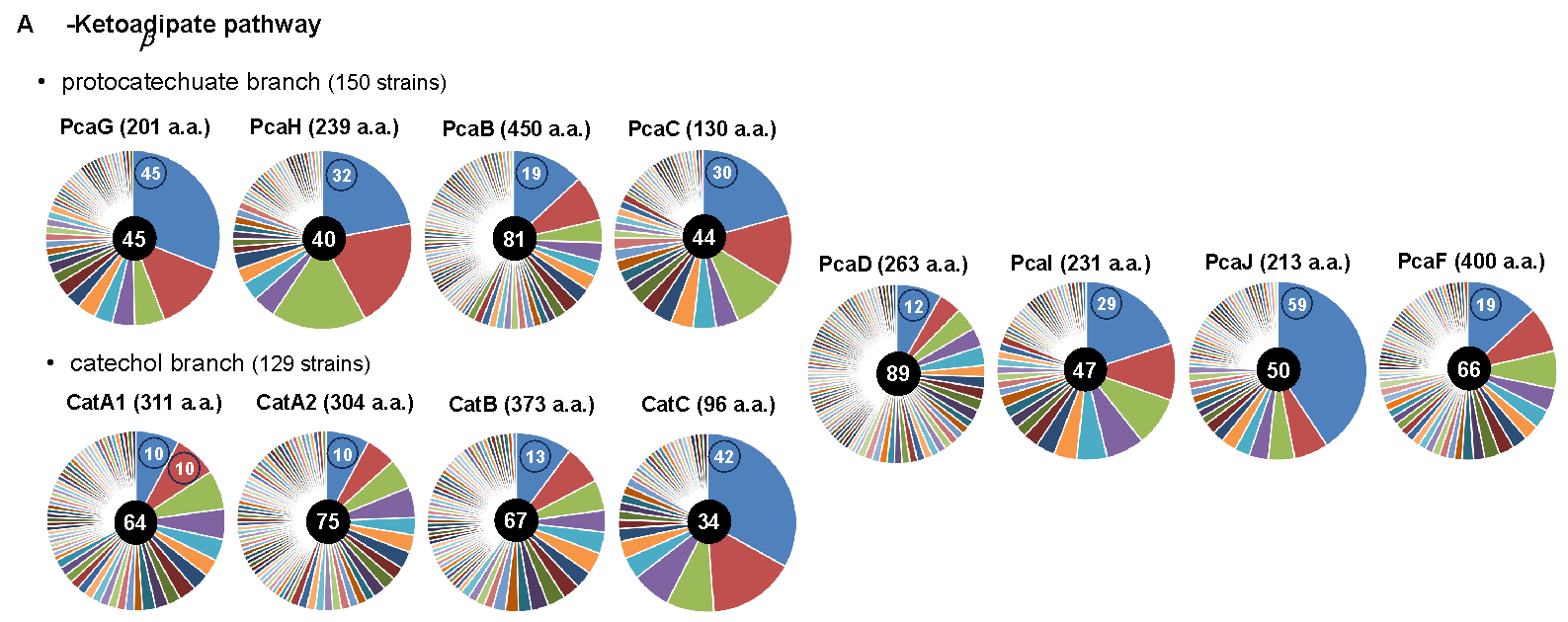


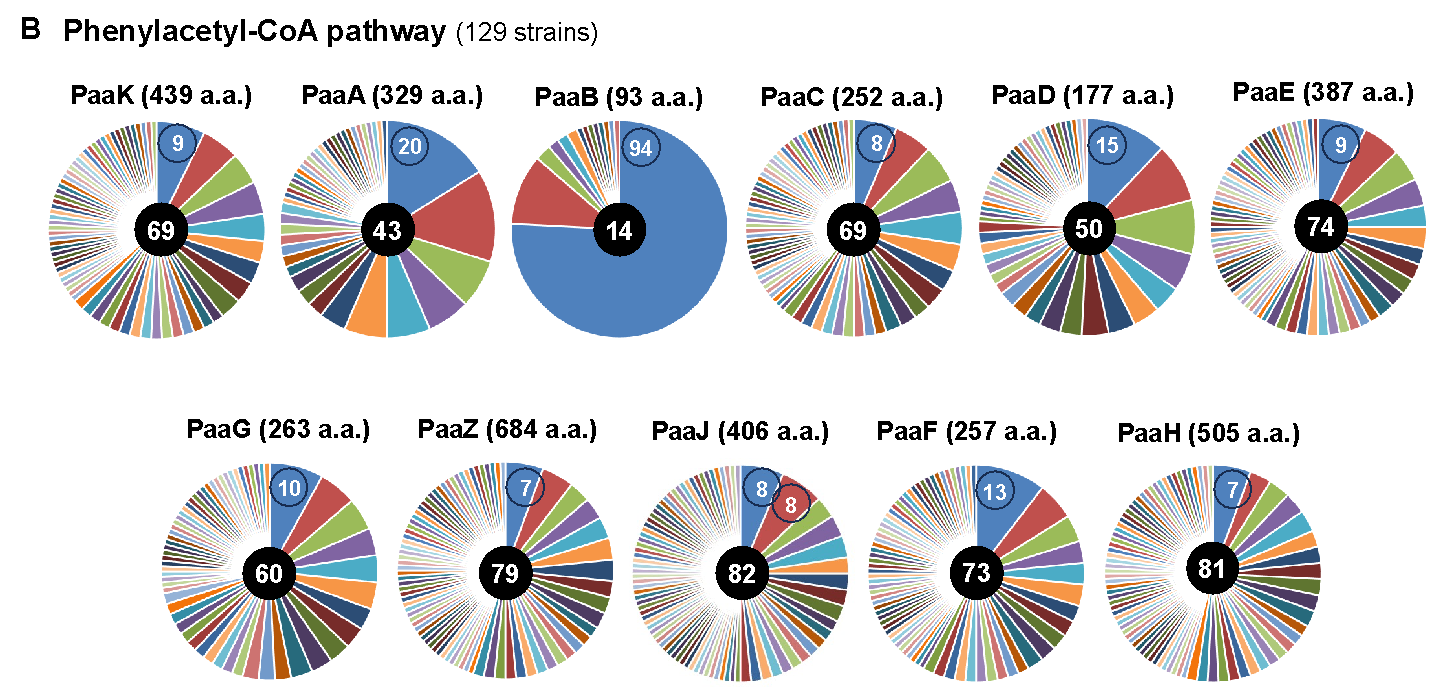


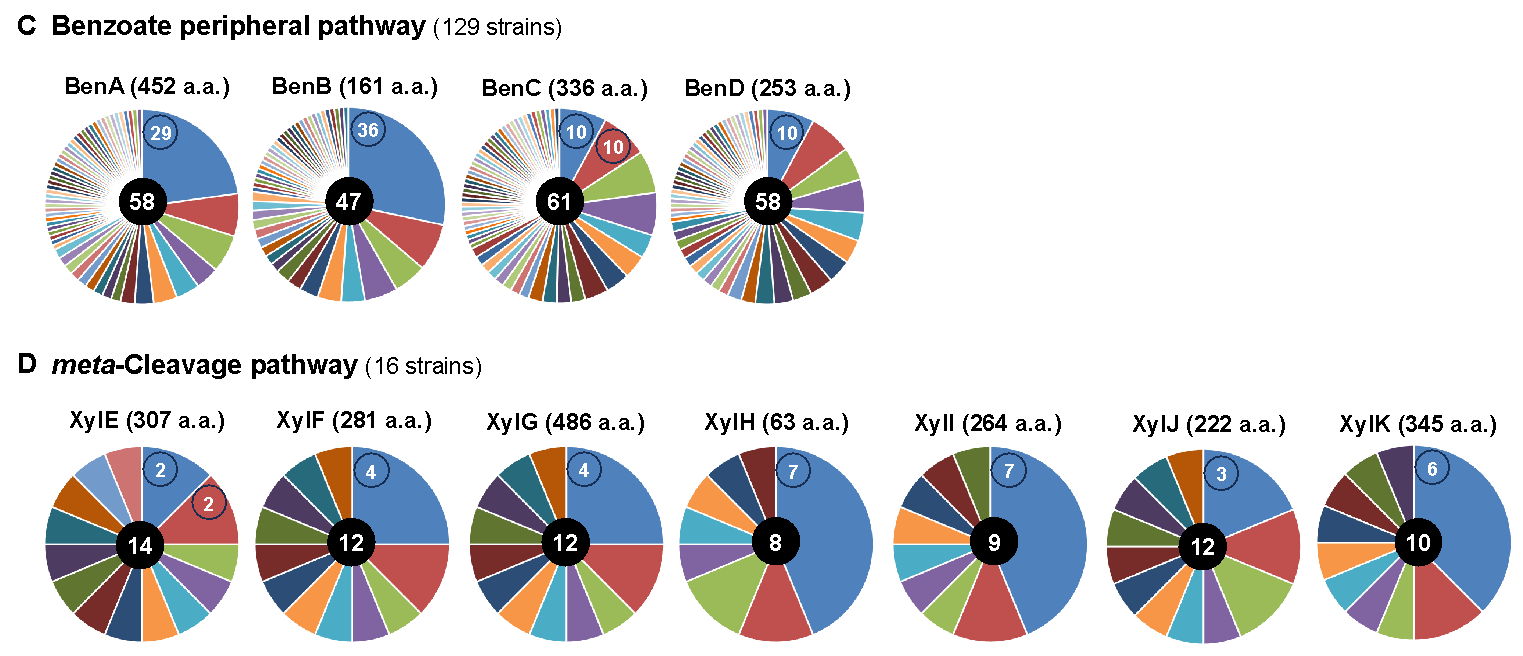


**Figure S8.** Alleleome analysis of pathway enzymes in aromatics metabolism. Alleleomes are plotted as pie charts based on the strain count of each allele, with values for the dominant alleles marked. Allele counts are shown at the center of the charts.


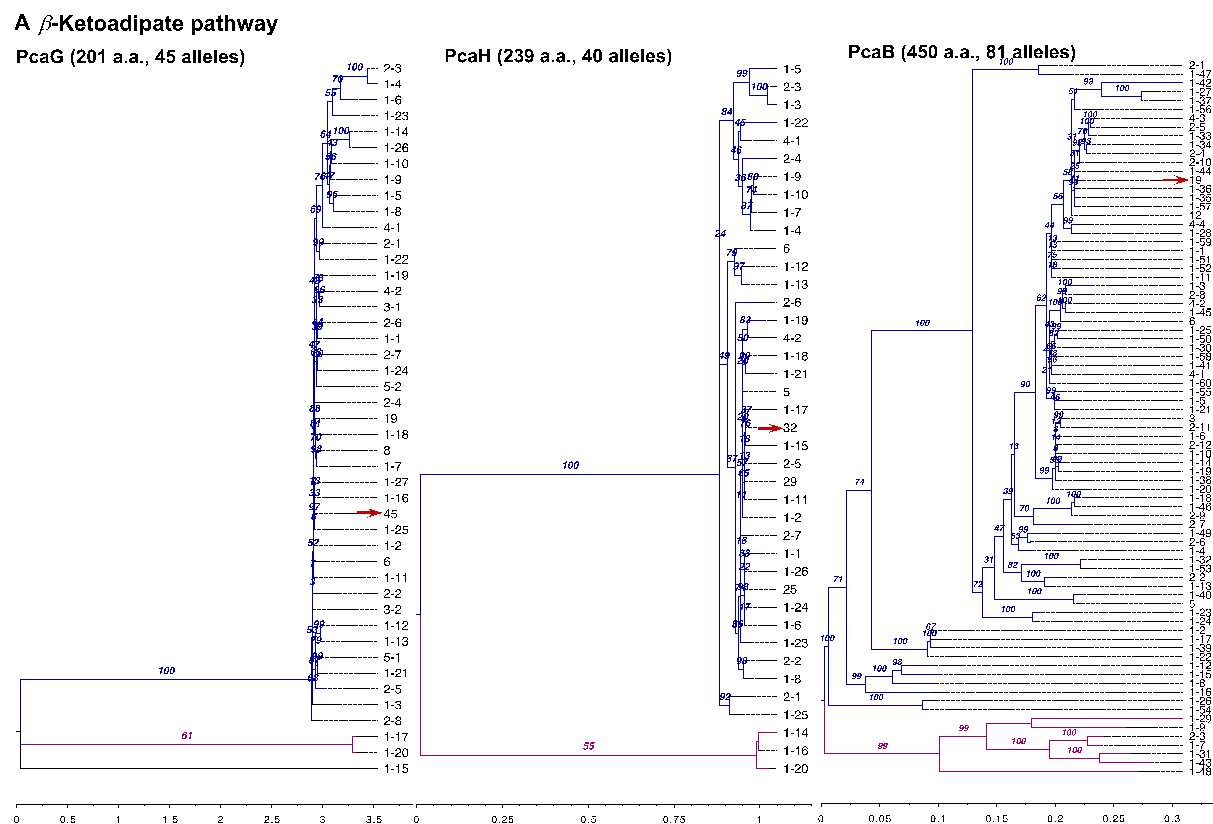

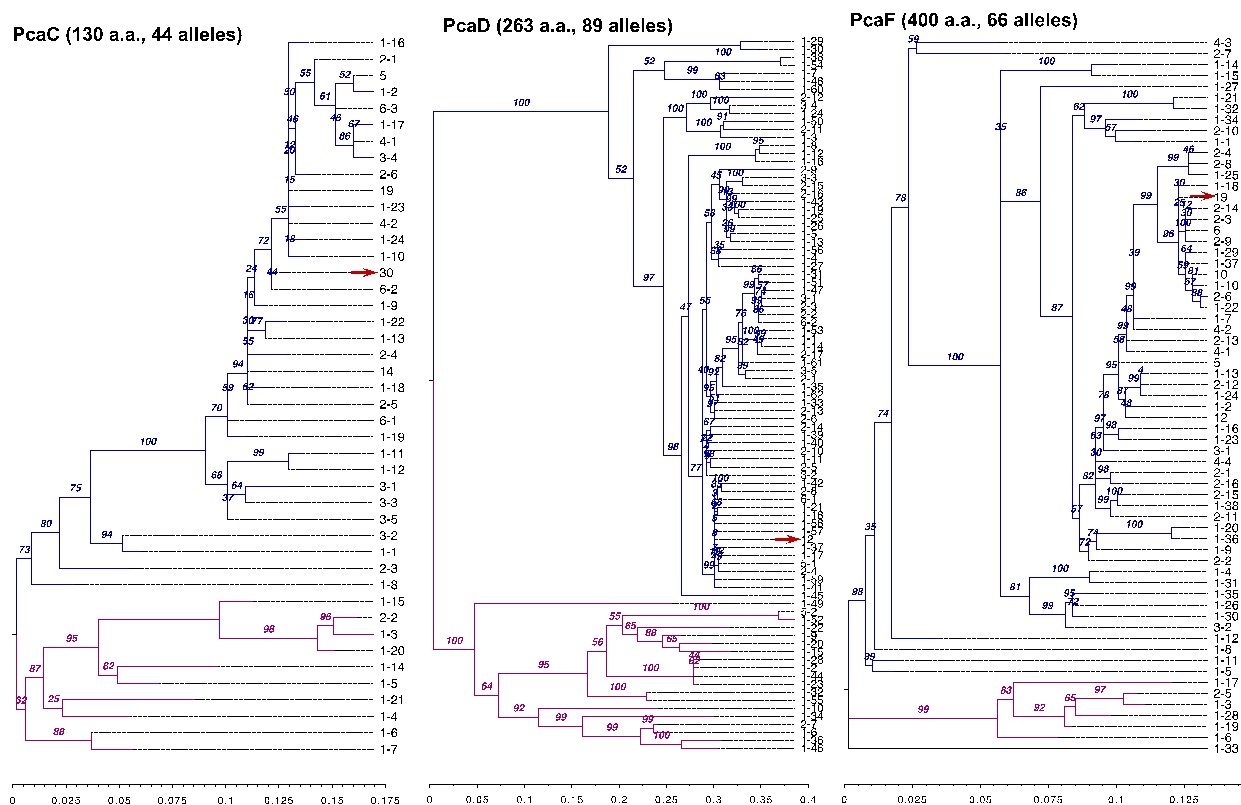


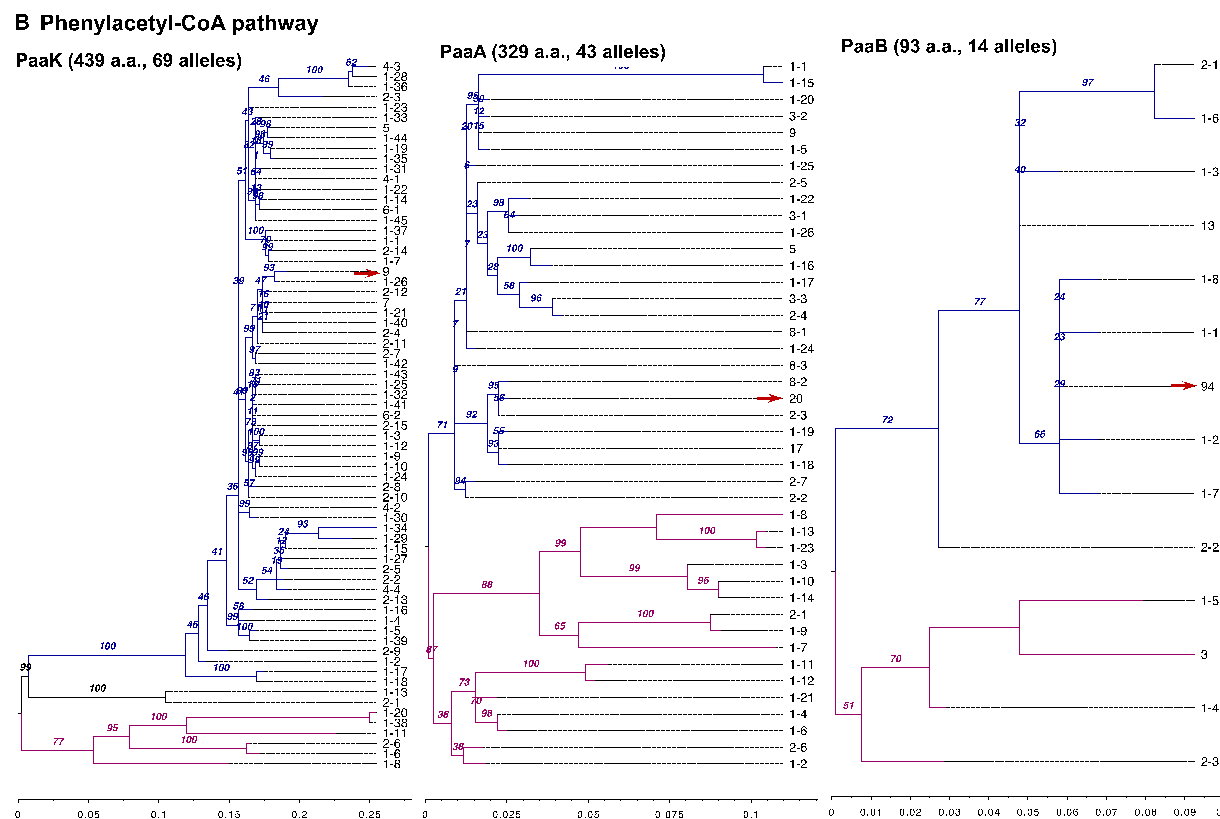

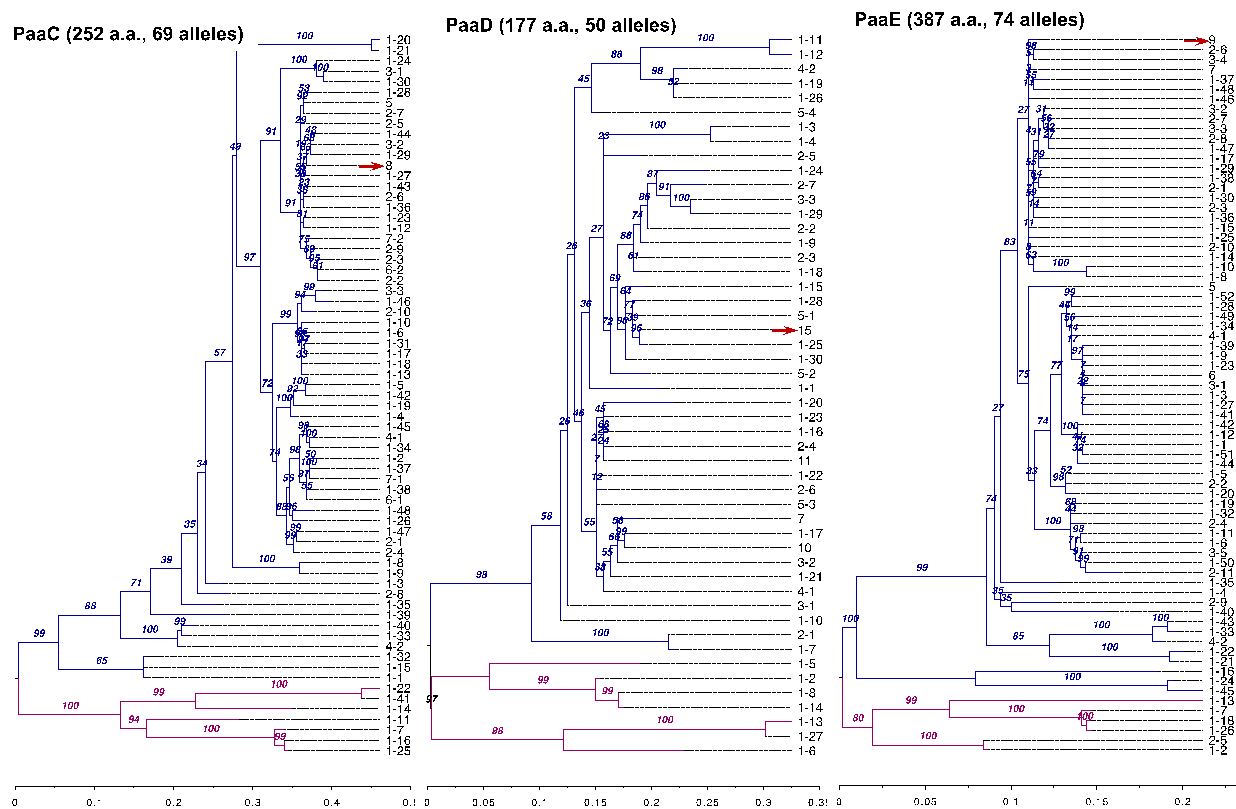


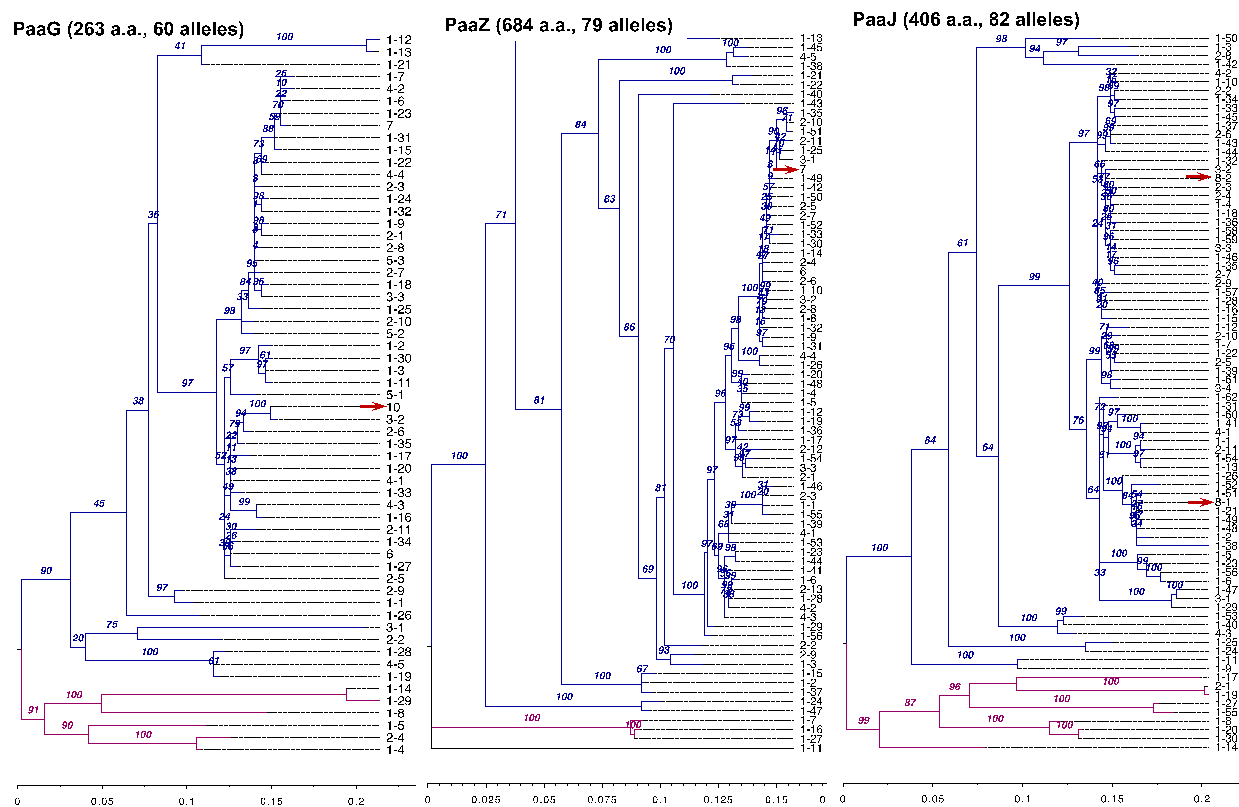


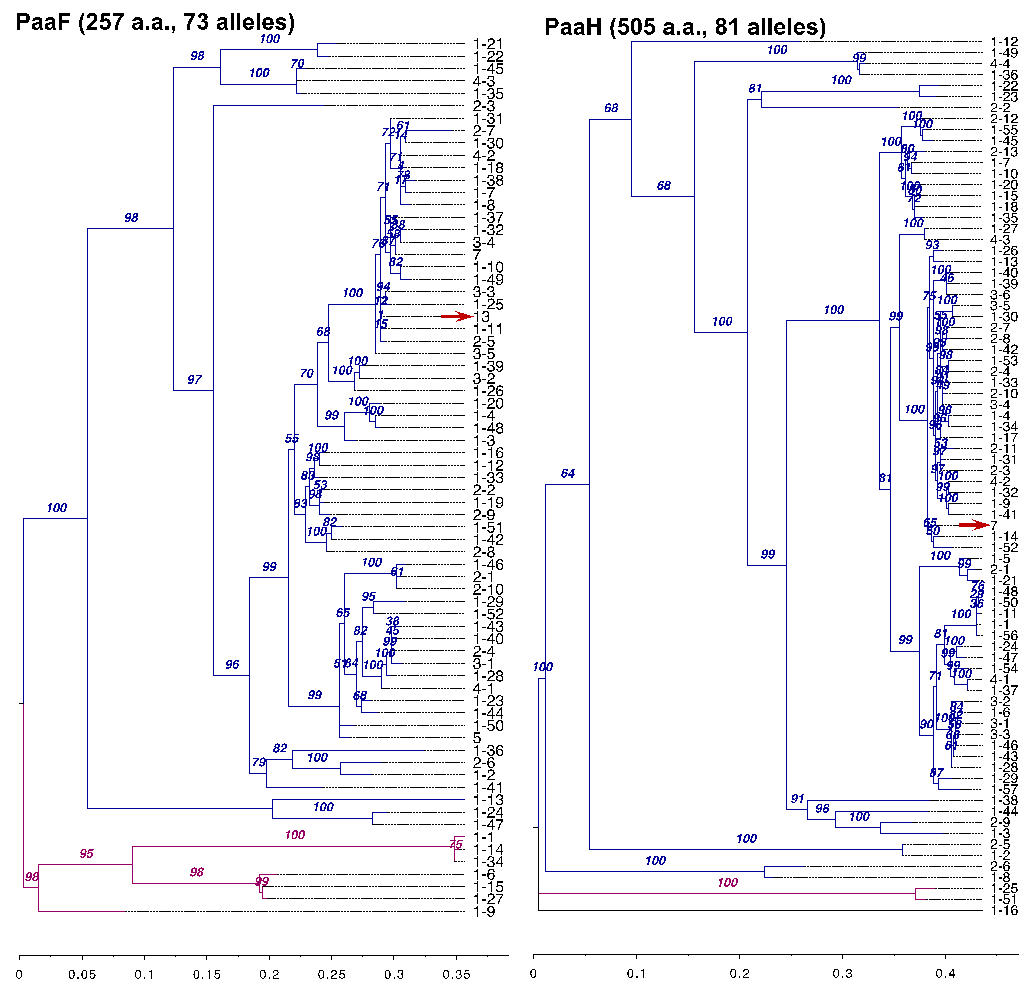


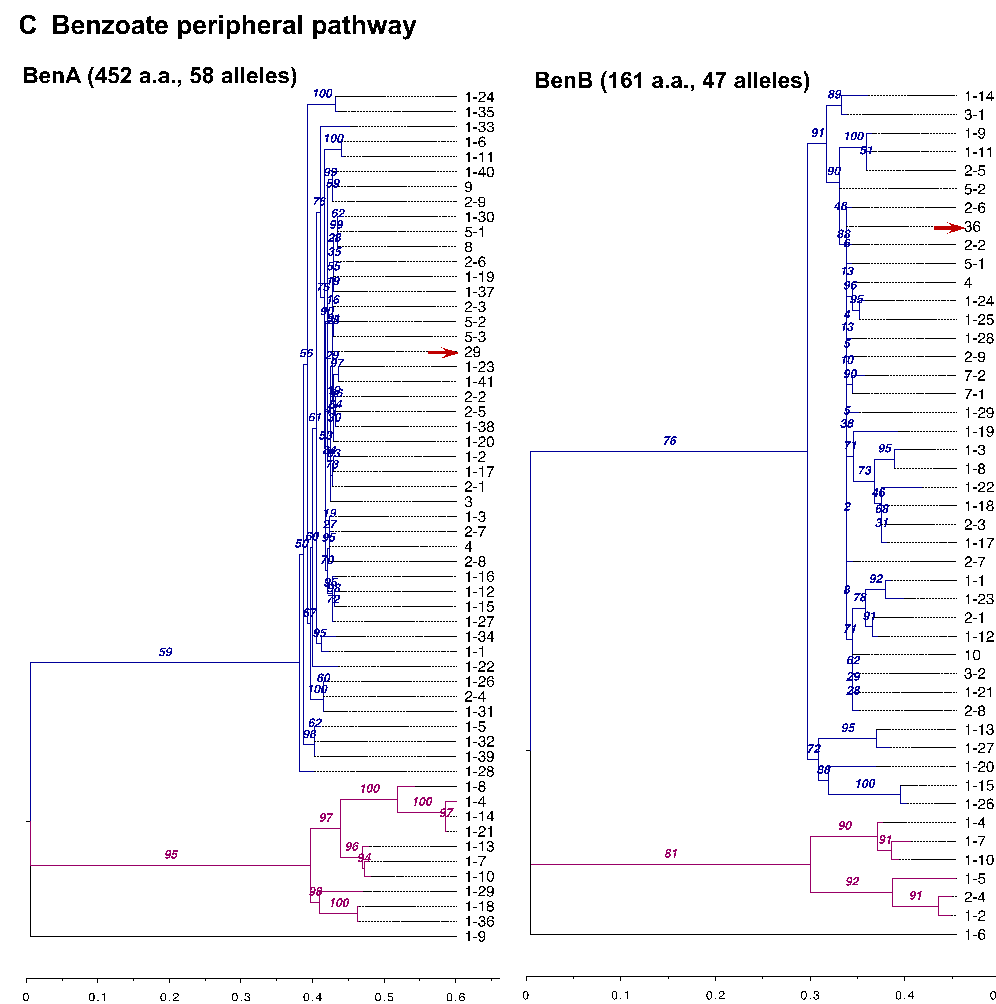


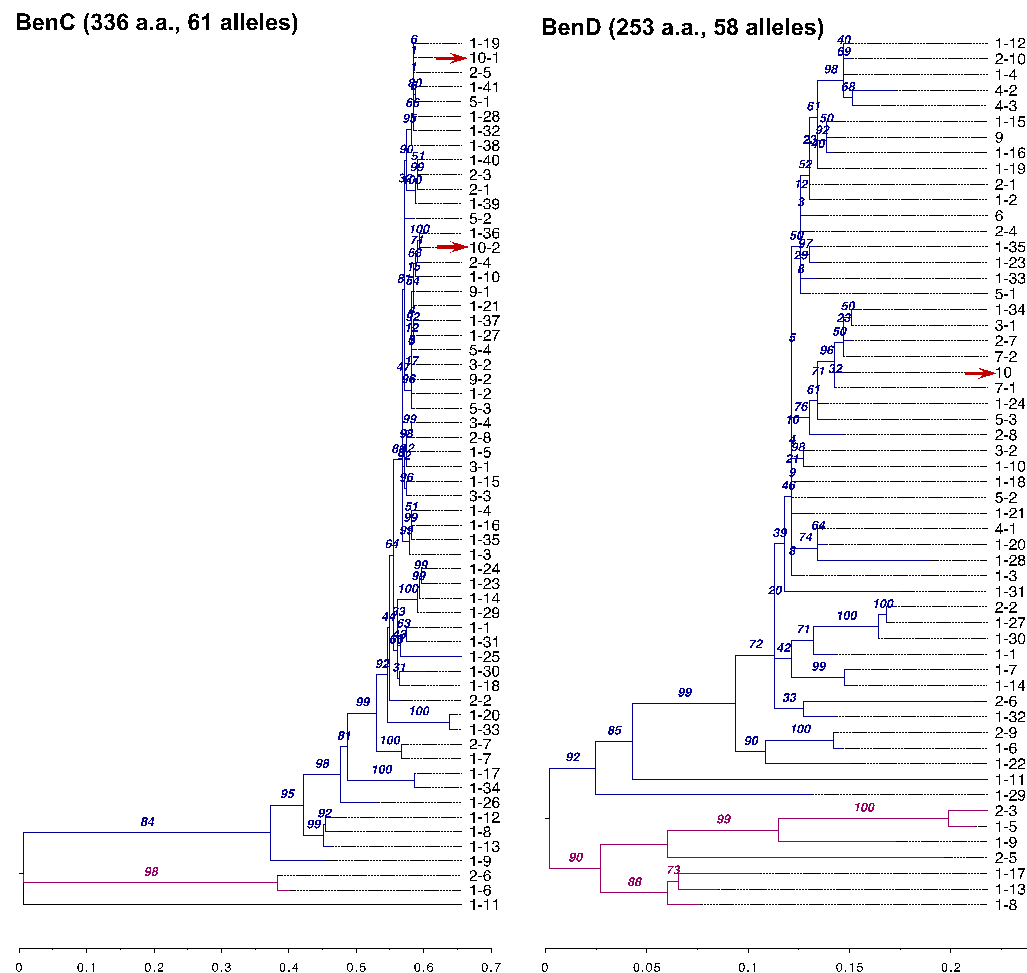


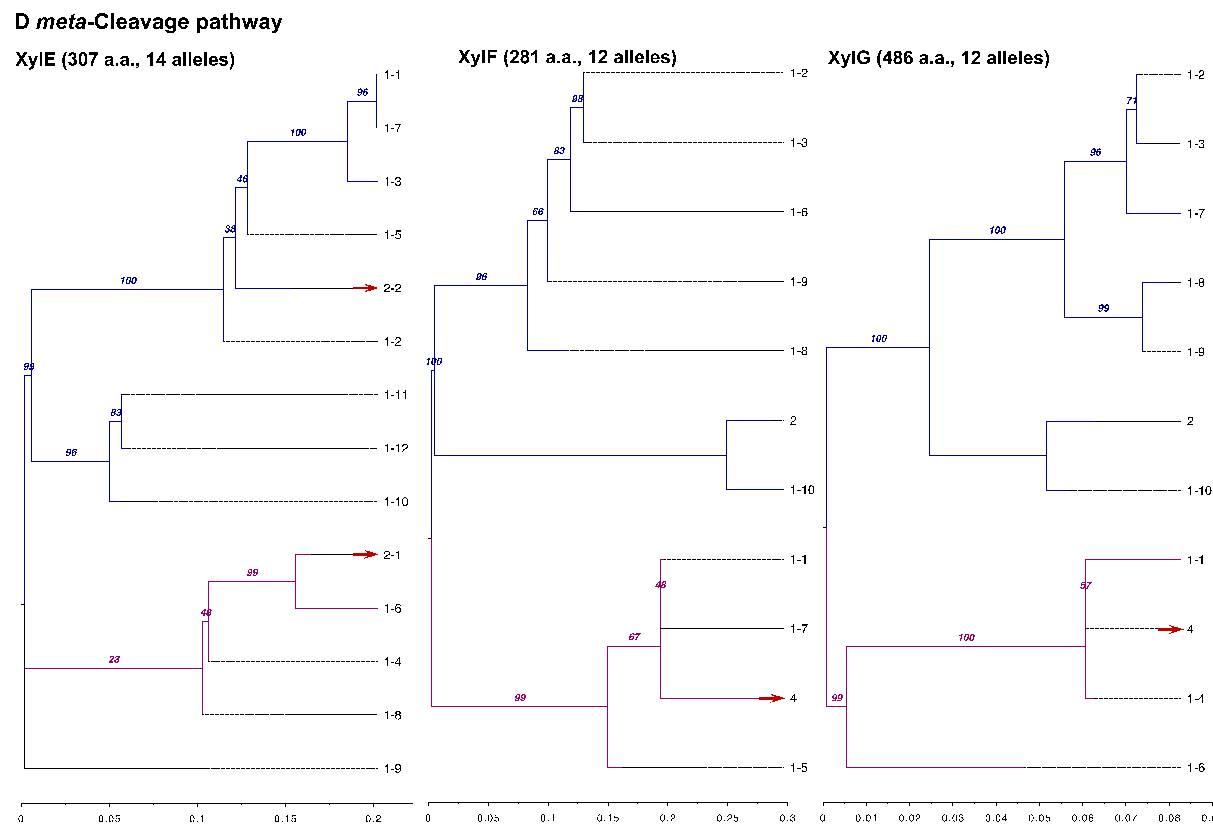

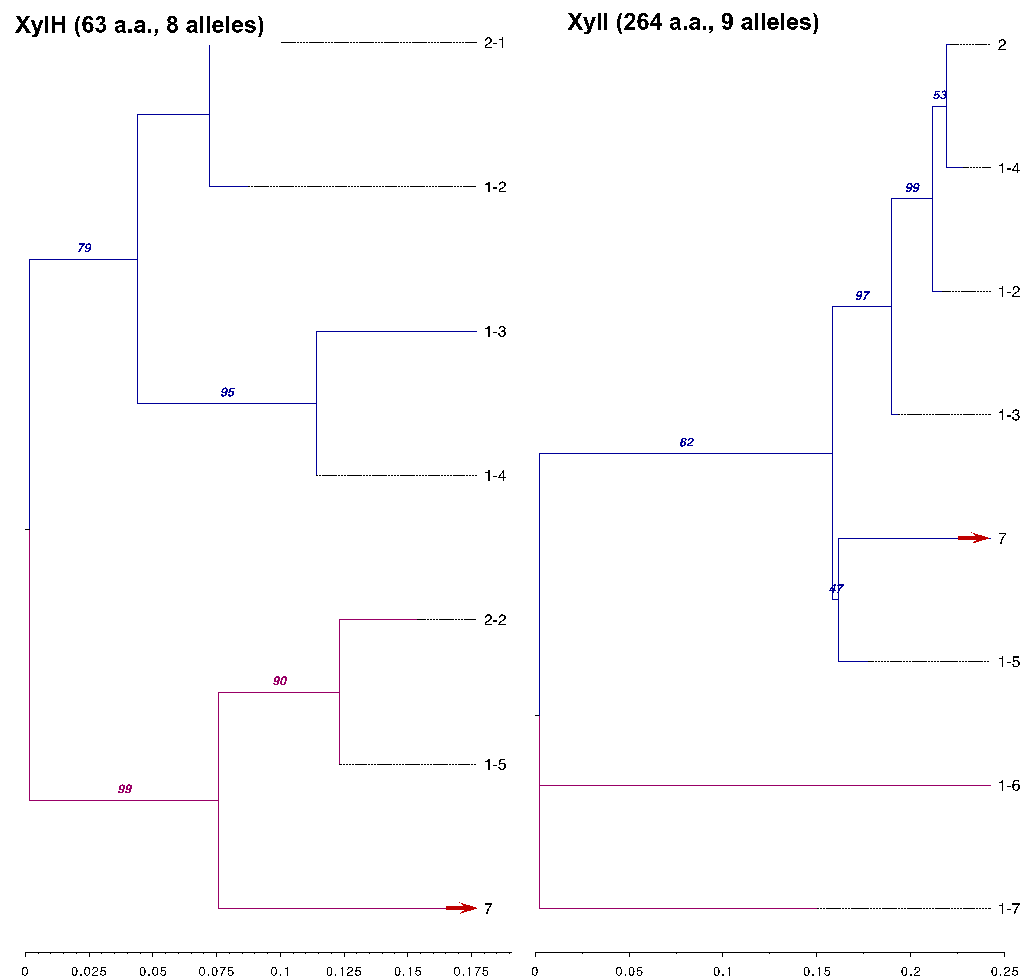

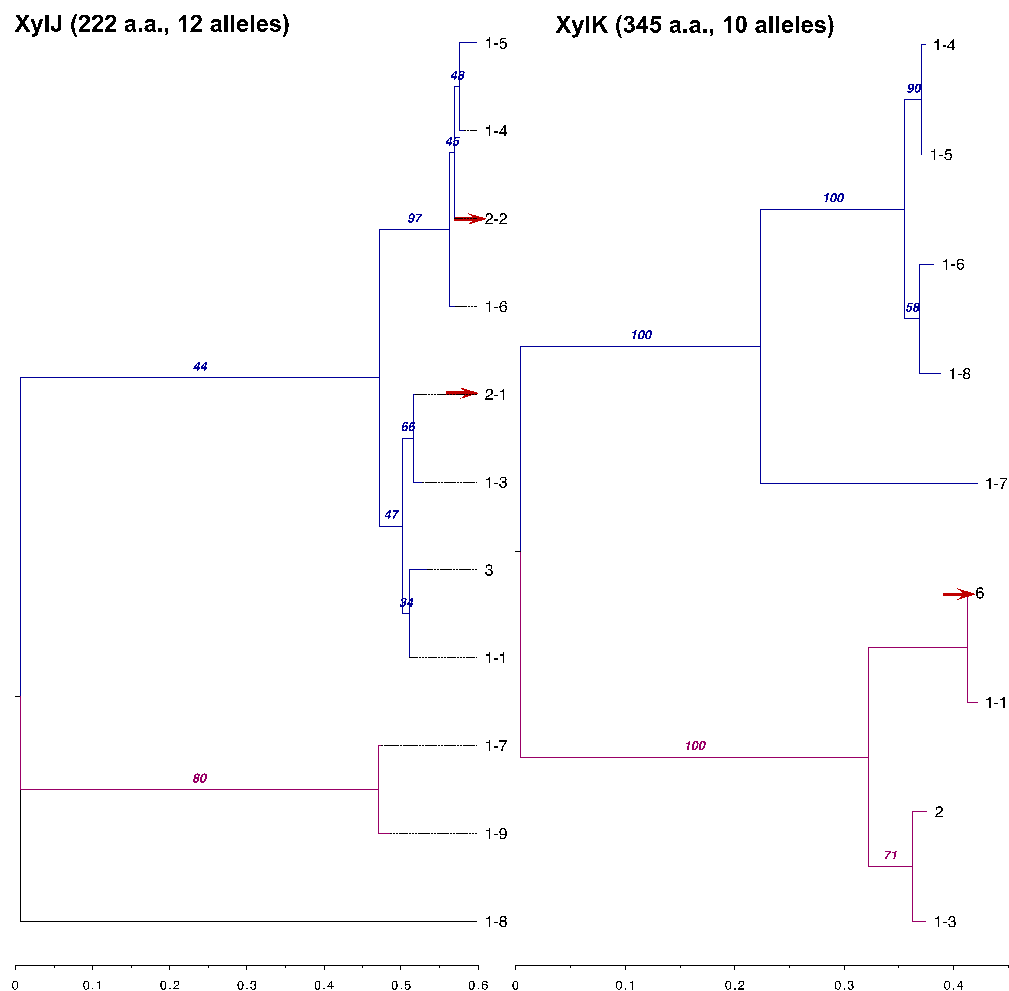


**Figure S9.** Phylogenetic analysis of pathway enzymes in aromatics metabolism using maximum likelihood method. Major clades are shown in blue, and minor clades are shown in purple. Alleles are named according to their strain counts. The corresponding strains are listed in Table S9. Dominant alleles are marked with asterisks. Scale bars represent the number of substitutions per site.
